# Polythiophene Synthesis Coupled to Quartz Crystal Microbalance and Raman Spectroscopy for Detecting Bacteria

**DOI:** 10.1007/s13758-012-0067-1

**Published:** 2012-11-06

**Authors:** R. P. Kengne-Momo, F. Lagarde, Ph. Daniel, J. F. Pilard, M. J. Durand, G. Thouand

**Affiliations:** 1 LUNAM Université, Institut des Molécules et Matériaux du Mans (IMMM), UMR CNRS 6283, Université du Maine, Avenue Olivier Messiaen, 72085 Le Mans Cedex, France; 2 LUNAM Université, Université de Nantes, UMR CNRS GEPEA 6144, IUT, 85035 La Roche Sur Yon, France; 3 Laboratoire de Chimie Organique, Université de Yaoundé I, B.P 812, Yaoundé, Cameroon

## Abstract

A simple electrochemical procedure was used for the synthesis of a polythiophene containing*para*-benzenesulfonyl chloride groups. The obtained polymer was shown to be very reactive and directly able to covalently bind nucleophile biomolecules. Protein A and a specific antibody were then successively immobilized on the conductive polymer through a covalent bonding of Protein A with the as-prepared linker for bacteria trapping purpose. All reactions were controlled in situ by cyclic voltammetry, quartz crystal microbalance and Raman spectroscopy. The results were compared to those previously obtained on gold surface modified with the same chemical linker. The conductive polymer led to a very high rate of antibody recognition compared to the gold surface and to literature, probably due to a large available surface obtained after polymerization. One example of pathogenic bacteria “*Salmonella enterica paratyphi”* detection was successfully tested on the substrates. The presented results are promising for the future design of simple and inexpensive immunocapture-based sensors.

## Résumé

Un polymère de type polythiophène comportant des groupements chlorure de parabenzenesulfonile a été synthétisé par voie électrochimique afin d’obtenir une surface destinée à l’immobilisation rapide et efficiente de biomolécules. Grace à une liaison sulfonamide, une protéine A a ainsi été fixée pour permettre l’accroche et l’orientation d’un anticorps spécifique. Le dispositif a pu être testé pour piéger une bactérie pathogène “*Salmonella enterica paratyphi”*. La présence de la bactérie sur la surface a été vérifiée conjointement par microbalance à quartz et par spectroscopie Raman. Les résultats obtenus ont été comparés à ceux obtenus sur une surface d’or fonctionnalisée selon le même procédé et ils témoignent de la très grande réactivité de la surface polymère. Ce procédé de fonctionnalisation de surface, facile à mettre en œuvre, spécifique et peu coûteux, constitue, par couplage avec la technique de diffusion Raman, une méthode alternative potentielle de détection et identification rapide de microorganismes pathogènes.

## Introduction

The biomolecule immobilization is one of the most important steps in the development of a new biosensor. Once immobilized, the biomolecules need to keep their original functionalities which means that a special attention must be taken so that the recognition sites are not sterically hindered [1]. A common reason for biosensor failure or underperformance is the chemical inactivation of the active recognition sites during the immobilization stages. For a biosensor to catch a bacterium for its identification, a common pattern is to first immobilize the specific antibody on the biosensor surface. But immobilizing biomacromolecules onto surfaces is a critical step, the surfaces are required to possess functional groups such as amine, imine, carboxyl, hydroxyl, isocyanate, epoxy, etc. [2]. In the case of antigen–antibody recognition, it is also very important to control the orientation of the biomolecules immobilized on the surface. Indeed, the recognition of an antigen requires the proper orientation of the specific antibody. Many studies showed that problems associated with the loss of biological activity upon immobilization of antibodies are noticeable in many cases. One of the main reasons for this reduction is attributed to the random orientation of the antibodies on support surfaces [3–5]. Thus, to elaborate a specific and highly sensitive immunosensor, the density and orientation of immobilized antibody are key factors. Numerous methods to bind antibodies to gold-coated transducers have been reported, among which the use of a binding protein appears to be attractive as it enables a favorable orientation of the antibody and thus leads to a satisfying biological activity of the sensor [6]. Among many others, Protein A is often used in this purpose [7, 8] because it is a cell wall component of *Staphylococcus aureus* that binds specifically to the Fc portion of IgG from many mammals [8, 9].

Consequently, the development of methods to assess the grafting of proteins on a surface has been the focus of considerable research activity in the recent years. Surface functionalization schemes, such as self-assembled monolayers (SAM) and phospholipid bilayer, make it possible to specifically immobilize proteins on surfaces, to control their surface density, to increase their orientation order and prevent them from denaturing [10, 11]. However, these techniques are time consuming, complex, and require hazardous or expensive materials, involving many experimental steps and chemicals.

In order to overcome some of the above limitations, the present paper presents a fast and efficient scheme of functionalization with*para*-benzene-sulfonyl chloride applied to a polythiophene surface. Since most conventional polymers have no such functional groups on the surface, they should be modified so as to have reactive groups suitable for covalent immobilization of biomacromolecules. The benzenesulfonyl chloride compounds are highly reactive to protein nitrogen groups and their reactivity has been previously used for purification of protein and nucleic acid, and to build bio-supramolecular structures. In this study, benzenesulfonyl chloride was electrochemically grafted on conducting polymer substrates. The conducting polythiophene derivative was synthesized with channel spacer and arylsulfonamide groups possessing an electrochemically and selectively cleavage S–N bond as described in literature [12, 13]. It was established that by applying a negative potential of about −2.5 V (vs. Ag/0.1 M Ag^+^), substituted amino moieties have been released from the polymer film covering the electrode surface into the electrolytic solution. The cathodic cleavage of the S–N bond was found to be favored by the conducting state of the polymer. Furthermore, the material could be regenerated after the cleavage step as a primary or secondary amine was easily anchored to the polymer without modifying its electrochemical properties. All parts of the process were monitored with a quartz crystal microbalance (QCM) and controlled by spectroscopic techniques.

The whole procedure allowed the formation of a reactive sulfonyl chloride layer on the polymer, on which Protein A and a specific IgG were then successively immobilized. The achieved results were compared to those previously obtained after similar immobilizations on a gold surface modified with the same reactive linker [14]. Both surfaces were finally tested for *Salmonella* detection.

## Materials and Methods

### Polythiophenes (PT) Synthesis

3-(oxyalkyl)-thiophene bearing arylsulfonamide group (*N*,*N*-diethyl-(2-thiophen-3-yl-ethoxidemethyl)-benzene-sulfonamide: C_17_H_23_NO_3_S_2_) was used as monomer. It was synthesized as described previously [12]. AT-cut 9 MHz platinum coated quartz crystal oscillators of platinum surface area 0.2 cm^2^ (Seiko EG&G) were used as working electrodes. The reference electrode was a silver wire immersed in 0.1 M AgNO_3_ and the counter electrode was a platinum wire. Tetrabutylammonium hexafluorophosphate (Bu_4_NPF_6_) from Fluka was used as support salt without further purification. The electrolytic solution was acetonitrile (purchased from Fluka) containing 0.1 M Bu_4_NPF_6_. Experiments were performed under an argon atmosphere and the electrolytic solution containing monomer was dried over neutral alumina (from Merck) activated at 300 °C under vacuum during 3 h. Electrochemical investigations were carried out in a one-compartment QCM cell in Teflon in order to follow in situ the mass fluctuations in comparison with the applied potential. The cell was connected to a VMP PAR Model VMP2/Z-40 potentiostat driven by the EC-Lab Software version 6.70. Microgravimetric measurements were performed by using AT-cut 9 MHz platinum coated quartz crystal oscillators connected to a PAR quartz crystal analyser model QCM 922 driven by the winEchem Seiko EG&G Software. After polymerization, the films were washed with acetonitrile and transferred into electrochemical cell containing only liquid electrolyte. Then, negative potential was applied (0 to −2.5 V) and the cell was connected to a VMP PAR Model VMP2/Z-40 potentiostat driven by the EC-Lab Software version 6.70. The liberation of amine from polymer was recorded by cyclic voltammetry with the potential scan rate of 100 mV s^−1^. The sulfinate ions obtained were then chlorinated by *N*-chlorosuccinimide in acetonitrile (3.4.10^−2^ M) at room temperature.

### Gold Surface Functionalization

Commercial QCM electrodes of gold surface area 0.2 cm^2^ were used as the substrates. The whole preparation procedure was already described in details [14]. Briefly,*para*-benzene-sulfonyl chloride functionalized QCM gold surfaces were prepared by electrochemical processes using sulfanilic acid (C_6_H_7_O_3_NS, Sigma-Aldrich) as the starting precursor. The reaction was performed at room temperature and pressure. Sulfanilic acid, 4.3 and 1.72 mg of sodium nitrite (NaNO_2_, Sigma-Aldrich) were dissolved in 5 ml of 1 M hydrochloric acid (HCl, Sigma-Aldrich). The mixture was introduced into the electrochemical cell constituted of a QCM gold substrate as work electrode, saturated calomel electrode as reference electrode and a platinum wire as counter electrode. Cyclic voltammetry experiment (Princeton applied research, VMP2) was performed in the potential range of 0.3 to −0.25 V at potential scan rate of 100 mV s^−1^ up to five cycles. After electrochemical reaction, the substrates were immersed in 5.2 mg phosphorous pentachloride (PCl_5_, Sigma-Aldrich)/5 ml dichloromethane (CH_2_Cl_2_, Sigma-Aldrich) solution. Finally, the substrates were washed in CH_2_Cl_2_ to remove unreacted PCl_5_.

### Protein and Antibody Immobilizations for Final Substrate Preparation

After chemical preparation of the substrates, staphylococcal Protein A (PrA, Sigma-Aldrich P 6031, 42 kDa) and a monoclonal antibody to *Salmonella* serogroups A, B, C1, C2, D, E1, E3, E4, F, G1, G2 (Mouse/IgG-2b named S-IgG, ACRIS AM05073PU-N, 155 kDa) were successively immobilized. Both biomolecule solution were prepared in phosphate buffered saline (PBS) solution at pH 7.4 with a concentration of 50 mg/l for PrA solution and 10 mg/l for S-IgG. The biomolecules were immobilized on the surfaces by consecutive immersions of the substrates in the solutions during 2 h each under QCM monitoring. After each step of immobilization, samples were washed in PBS and water and dried at room temperature for Raman analysis. According to ACRIS compagny, the antibody S-IgG is specific for detection of *Salmonella*, no cross reaction have been noted with other bacteria (*Escherichia coli*, *Klebsiella*, *Pseudomonas,* etc.).

### Fluorescent Antibody and Bacteria Solutions for Sensor Evaluation

In order to assess the anchoring of both biomolecules, a fluorescent polyclonal antibody to mouse IgG(H&L)-FITC (F-IgG, Acris R1253F, 155 kDa), thereafter named F-IgG, was prepared at a concentration of 140 mg/l in PBS and both as-prepared substrates were immersed in this solution for 2 h prior QCM and fluorescence microscopy analysis.

To test the as-prepared substrates for biodetection capabilities, bacteria *Salmonella enterica paratyphi B* were cultivated in 10 ml of peptone middle overnight at 30 °C under stirring at 250 rpm. Then, the bacteria solution was washed three times in phosphate buffer saline and its optical density (measured at a 620 nm wavelength) was adjusted to 0.1. Both final substrates with grafted PrA and S-IgG were immersed in the bacteria solution during 2 h under QCM recording and, after washing, the surfaces were analyzed by Raman spectroscopy.

### Raman Spectroscopy

Raman spectroscopic measurements were conducted using a confocal laser Raman spectrometer (Xplora, Horiba). The Raman signals were recorded in a spectral range 400–3,100 cm^−1^ with an integration time of 10 s using a 785 nm laser excitation wavelength and a power not exceeding 30 mW, in combination with a 100× objective of an Olympus BX41 microscope. All the raw data were then treated with standard OPUS (Bruker Optics software) software for linear baseline correction, smoothing and min–max normalization.

### Quartz Crystal Microbalance (QCM) Measurements

In order to monitor the biomolecule immobilization on the surfaces, QCM measurements were performed by using AT-cut 9 MHz platinum coated quartz crystal oscillators connected to a PAR quartz crystal analyser model QCM922 driven by the winEchem Seiko EG&G Software. Frequency changes were monitored in electrochemical cells. 200 μl of PBS was initially injected into the cell and the background frequency was stabilized during several minutes. Frequency changes for biomolecule immobilization were then measured by injecting 10 μl of biomolecule solution into the cell. In the first step, PrA solution was injected and the frequency changes were monitored. After that, the platinum surfaces were washed in PBS and water before continuing the process of immobilization. Then, the solution of antibody (S-IgG) and bacteria were successively injected into the cell.

For quantitative estimation of the process, the successive coated masses were re-calculated (in ng) from the frequency shifts using Sauerbrey equation [15].

In this equation: ΔF (Hz) is the frequency change, Δm (g) is the added mass, F_0_ is the fundamental resonant frequency of unloaded quartz (9 MHz), μ_Q_ is the shear modulus of AT-cut quartz (2.947 × 10^11^ g cm^−1^ s^−2^), ρ_Q_ is the density of the quartz (2.648 g cm^−3^), A is the surface area (0.2 cm^2^).

This equation establishes that the decrease in frequency is linearly proportional to the increase in surface mass loading on QCM when the adsorbed films can be considered as uniform, rigid and ultra thin (less than 15 μg in this study with this equipment). Here, the gold surface, the polymer film and the monolayer surfaces were roughly considered as rigid. The potential viscoelasticity of the antibody layers was neglected in order to be able to apply the Sauerbrey equation. Thus, the calculated Δm (ng)/ΔF (Hz) ratio = −1.066 was considered as an acceptable approximation for PrA and IgG immobilizations, but was not used to quantify bacteria immobilization.

### Fluorescence Microscopy

Fluorescence measurements were recorded using an Axio Scope microscope (Carl Zeiss Microimaging GmbH, Germany) driven by axiovision Rel. 4.8 software. The mercury-vapor lamp for reflected light illumination was used. The fluorescence images were collected using axiocam ICc camera, with the filter set for reflector module at the position corresponding to fluorescein isothiocyanate (FITC) fluorophore. The excitation and emission wavelength of this fluorophore are respectively 495 and 520 nm. The 40× objective with a numerical aperture equal to 0.75 was used. All experiments were done in dark room to avoid external contribution.

## Results and Discussion

### Polymer Film Preparation

**Fig. 1:**
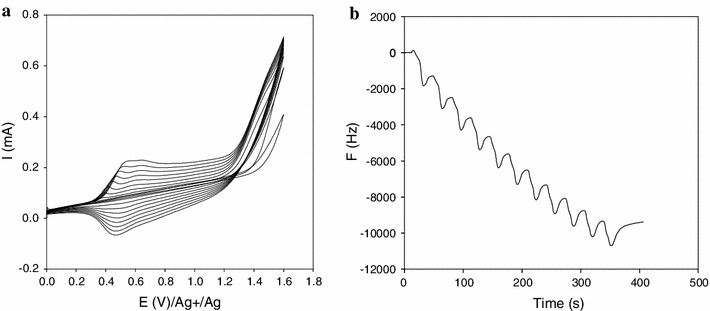
**a** Cyclic voltammograms at 100 mV s^−1^ of a polythiophene in 0.1 M Bu_4_NPF + CH_3_CN; **b** variation of frequency occurring during polymerization

Figure 1a shows the cyclic voltammograms recorded during polymerization in the range 0 to +1.5 V. The first sweep presents a wave centered at around 1.35 V corresponding to the oxidation of the thiophene monomer. The next cycle presents a new redox system centered at 0.55 V. This system shows an increase with the number of sweeps. The reversibility of such systems corresponds to the p doping process of the conducting polymer which gradually coats the electrode surface.

**Fig. 2:**
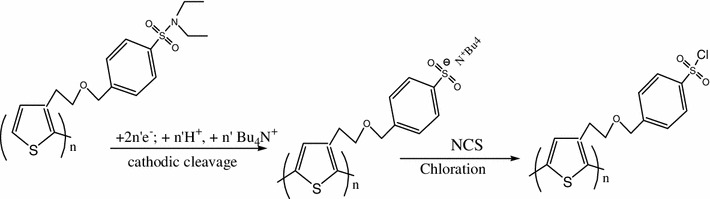
Electrochemical procedure for polymer activation

The corresponding variations of frequency occurring during deposition of the polymer film and monitored by QCM are displayed on Fig. 1b. The total frequency change was estimated at 11,000 Hz corresponding to a calculated mass of 11,726 ng using Sauerbrey equation. During this variation, an oscillated behavior corresponding to each electrochemical cycle was observed; it could be interpreted as follow: on the forward scanning, a mass was gained due to the entrance of PF_6_^−^ anions within the polymer chains for charges balance. The mass loss observed in the return scanning is attributed to the release of these PF_6_^−^ anions. The overall mass gain detected during the electrochemical process corresponds to the formation of the polymer layer. In order to activate the linker groups of poly [3-(oxyalkyl)-thiophene bearing arylsulfonamide group] synthesized, the film was then modified. Hence, the sulfonamide terminal functions of the started poly [3-(oxyalkyl)-thiophene bearing arylsulfonamide group] were electrochemically split and were furthermore chemically modified with *N*-chlorosuccinimide (NCS) leading to the expected sulfonyle chloride groups as illustrated on Fig. 2.

**Fig. 3:**
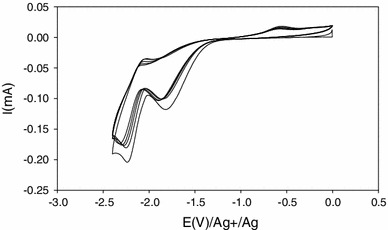
Voltammograms of cathodic scission of S–N bond in polythiophene at 100 mV s^−1^

Figure 3 shows voltammograms recorded during the S–N cleavage. As already reported by Dubey et al. [13], this figure displays a cathodic peak corresponding to the cleavage of S–N bond at −2.45 V. It has been demonstrated that, in order to preserve the electroactivity of the polymer film after cleavage, the cathodic potential should not be more negative than −2.55 V.

### Immobilization of PrA and S-IgG on Polythiophene and Gold Modified Surfaces

#### QCM Results

**Fig. 4:**
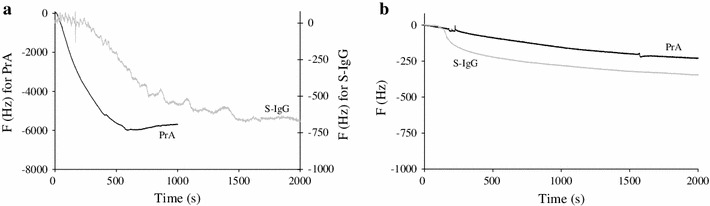
Quartz crystal microbalance responses to PrA and S-IgG immobilizations on **a** polythiophene surface and on **b** modified gold surface

For the successive immobilizations of PrA (50 kDa) and S-IgG (150 kDa) on polythiophene and gold modified surfaces, QCM responses are shown on Fig. 4. The recorded frequency variations for PrA were around 5,600 Hz (calculated mass of 5,969 ng) on functionalized polythiophene (PT) and 225 Hz (calculated mass of 240 ng) on the gold substrate. This huge difference of immobilized masses and the fastness of the reaction in the case of PT may be due to a very high available surface area provided by polymerization. Moreover, the roughness of the polymer surface may have led to adsorption phenomena while on pure gold, protein adsorption was undetectable by QCM [14].

However, this high amount of immobilized PrA did not led to a significant increase of the S-IgG immobilization compared to gold substrate. Indeed, when compared, the frequency variations recorded on both surfaces during the S-IgG immobilization process exhibit on PT (600 Hz corresponding to 639 ng) less than twice more immobilized S-IgG compared to gold (373 Hz corresponding to 397 ng). The ratio (given in Table 1) between S-IgG and PrA concentrations (S-IgG/PrA) increases from 1/33 on PT to 1/2 on gold. This means that roughly it needs 33 PrA on PT and 2 on gold to react with an S-IgG. The binding capacity on the gold surface is in full agreement with the literature which gives a 0.2–1.0 range [16, 17]. The low ratio of S-IgG immobilized on PT despite the high amount of PrA can probably be explained by sterical hindrance of active sites of PrA [18]. Hence, when one S-IgG reacts with the first site of PrA, the nearest site is hidden and leads to the global weak immobilization ratio of S-IgG on PT compared to the high coverage yield of PrA. In both cases, the injection of S-IgG solution led to sharp mass gains suggesting the high specific binding of PrA and S-IgG. In the case of non-specific interactions, a much higher immobilization of S-IgG would have been measured.

**Table 1:** Estimation of the S-IgG/PrA and F-IgG/IgG ratios immobilized on both surfaces

	S-IgG/PrA	F-IgG/S-IgG
Polythiophene surface	0.03	1.8
Gold modified surface	0.45	0.8

#### Raman Results

**Fig. 5:**
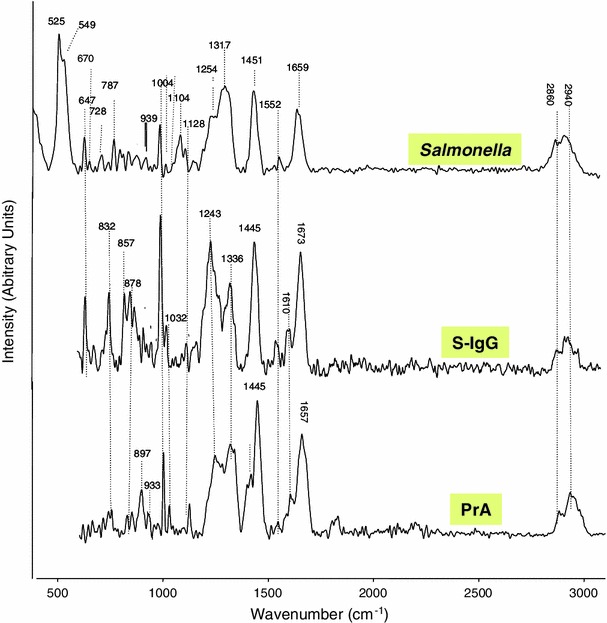
Raman spectra (785 nm, 10 mW) of PrA and S-IgG powder samples and of *Salmonella* bacteria (spectra of a pure *Salmonella* colony deposited on a gold surface)

**Fig. 6:**
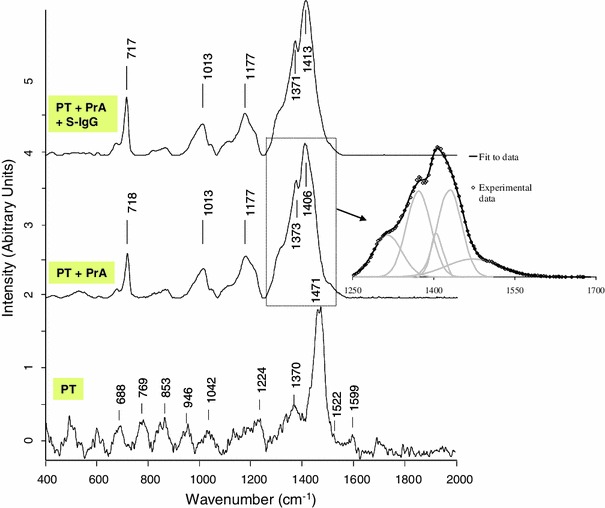
Raman spectra (785 nm, 10 mW) of functionalized polythiophene; PrA and S-IgG immobilized on functionalized polythiophene. The detail concerning the data fit performed for the region 1,200–1,700 cm^−1^ is related to the PT + PrA spectrum

**Fig. 7:**
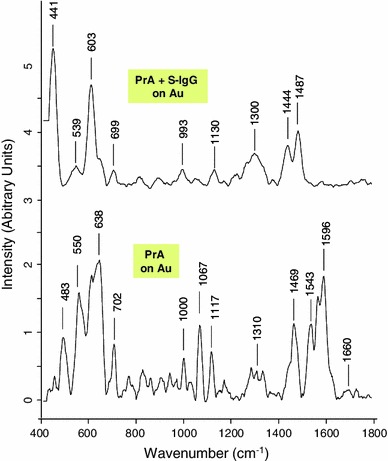
Raman spectra (785 nm, 10 mW) of PrA and S-IgG consecutively immobilized on functionalized gold surface

For comparison and discussion purposes, the spectra of PrA and S-IgG powders and the spectrum of a pure Salmonella colony are displayed on Fig. 5. The Raman spectra of PrA and S-IgG after immobilizations are exhibited on Fig. 6 for the polythiophene substrate and on Fig. 7 for the gold one.

**Fig. 8:**
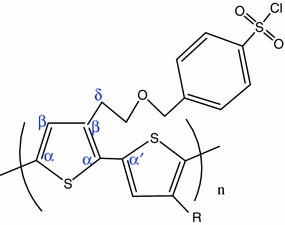
Structure of the synthesized polythiophene

The wavenumbers of the observed bands for the PT spectrum were assigned on the basis of references [19–21]. As stated in these references, and considering the structure of the synthesized polythiophene (Fig. 8), bands assignment could be made as follow: the most intense band at 1,471 cm^−1^ was attributed to the symmetric stretching mode of the aromatic (Cα=C_β_) bond ring. The band at 1,521 cm^−1^ was assigned to the antisymmetric stretching mode of the (Cα=C_β_) bond ring. It has been demonstrated that this antisymmetric stretching mode of the C==C band is of particular interest in the studying of conjugation length of the polymeric chain [12]. Hence, its position slightly shifts towards high frequencies, when the conjugation length of the polymer is increased. The band at 1,370 cm^−1^ was attributed to (C_β_–C_β_) intra-ring symmetric stretching vibration and the band at 1,224 cm^−1^ was attributed to the inter-ring Cα–Cα′ stretching. The band at 1,042 cm^−1^ was attributed to the symmetric C–H bending. The bands at 853 and 946 cm^−1^ were attributed to the stretching vibration of C_β_–C_δ_. The bands at between 688 and 769 cm^−1^ could be assigned to the C–S–C deformations vibrations of thiophene rings. Finally, the slight 1,599 cm^−1^ peak was attributed to the benzene ring vibration mode of the*para*-benzene-sulphonyl chloride group [14, 22].

Then, by comparing the spectrum of PT with grafted PrA and the one of PT before grafting (Fig. 6), an intensity enhancement coupled with a slight shift towards lower wavelength of the most PT intense band was observed. The intensity enhancement (intensity approximately 1,000× compared to the raw polythiophene) does not appear on the Figure because, for a better comparison, the spectra were normalised. This kind of phenomena was already described in literature for neutral polythiophene after doping [23] and may confirm a strong modification of the polythiophene environment due to the presence of the proteins. To get a better understanding of the phenomena in the region 1,250–1,700 cm^−1^, the spectra were fitted in this frequency range with a standard fitting procedure (software Origin 6.0) and assuming that the shape of the Raman lines are roughly Gaussian. A detail of the fit obtained for (PT + PrA) band centered at 1,406 cm^−1^ is added on Fig. 6. It is shown that, to be correctly described, this broad block of Raman peaks has to be deconvoluted in 5 contributions located respectively at 1,312, 1,372, 1,406, 1,433 and 1,478 cm^−1^. For Raman analysis of proteins, many band assignments have been established throughout the years [24–28]; the most frequently reported ones are given in Table 2. By comparing with the Protein A spectra (Fig. 5) and to literature [24–28], it can be concluded that the band located at 1,312 cm^−1^ can be attributed to the Amide III of the protein while the 1,406 and 1,433 cm^−1^ could be assigned to CH_2_ bending contributions of the protein. However the 1,372 and 1,478 cm^−1^ components of this broad band are typically due to the polythiophene. However except this observation, it was quite difficult to clearly distinct the protein bands on the spectrum. This is probably due to the high intensity of the polythiophene, which led to “erase” the protein contributions.

**Table 2:** Assignment of some bands frequently found in Raman spectra of proteins [24–28]

Band frequency (cm^−1^)	Assignment
520–540	S–S stretching
640–829–852	Tyrosine
720	Adenine
880–895	CH_2_ rocking
1,002	Phenylalanine
1,061–1,129	C–N and C–C stretching
1,270–1,310	Amide III
1,340–1,345	Marker of *α*-*helix* conformation
1,440–1,460	CH_2_ deformation
1,553	Tryptophan
1,575	Guanine, adenine (ring stretching)
1,606	Phenylalanine
1,650–1,680	Amide I

Moreover, as already commented, the Raman spectrum of the synthesized PT exhibited many bands in the usual window (500–1,800 cm^−1^) of Raman characteristic peaks of proteins. Additionally the main characteristic bands of polythiophene are broad which made difficult an easy distinction with the bands of the protein. The band at 1,177 cm^−1^ does not corresponds exactly either to the protein, or to the polymer references; because this band is broad, it could be assigned as a combination or an overlapping of both protein and polythiophene signals. Only the bands at 1,013 and 718 cm^−1^ may indicate the respective presences of phenylalanine and adenine from PrA.

After S-IgG immobilization, the most intense band of PT (at 1,471 cm^−1^ originally) was once again slightly shifted towards a lower position (1,413 cm^−1^) but no other clear signal could assess the presence of the antibody on the surface.

On the contrary, as illustrated on Fig. 7, both PrA and S-IgG presences could be clearly distinguished on the modified gold surface. Briefly, the amino-acids—tyrosine, phenylalanine and tryptophan—were respectively identified at 638, 1,000 and 1,543 cm^−1^. The C–H deformation (δ C–H) band appeared at 1,469 cm^−1^. The α-helix structure of the Amide III was identified in the 1,270–1,345 cm^−1^ range [24, 28]. The 1,117 and 1,067 cm^−1^ bands were assigned to the backbone structure C–C stretching and the stretching of C–N bonds. On this surface, adenine appeared at 702 cm^−1^ and the band at 550 cm^−1^ could be attributed to the S–S stretching bond.

The spectra of immobilized S-IgG featured also the C–H deformation band of fat acids at 1,444 cm^−1^. The Amid III band appeared around 1,300 cm^−1^, tyrosine was identified in the 600–700 cm^−1^ spectral region and phenylalanine band appeared at 993 cm^−1^. The well resolved bands at 441 and 550 cm^−1^ could be assigned respectively to COC glycosidic ring deformation and S–S stretching bond. To conclude, many of the characteristic peaks of PrA and S-IgG were identified after immobilization on functionalized gold surface with little shifts suggesting that they conserved their native structure during immobilization; these shifts being the consequences of changes in the environment of the biomolecules constitutive groups. As Raman intensity is directly related to molecule polarisability and depends of the angle between the scattered angle and the dipolar moment associated to the concerned band, a change of orientation of a molecule can lead to a strong intensity variation for one band. The amide I band of Protein A is a good illustration of this phenomenon because it is a quite intense band on the Protein A spectra (Fig. 5) and it becomes very weak when the protein is linked to the gold surface.

### Immobilization of F-IgG

**Fig. 9:**
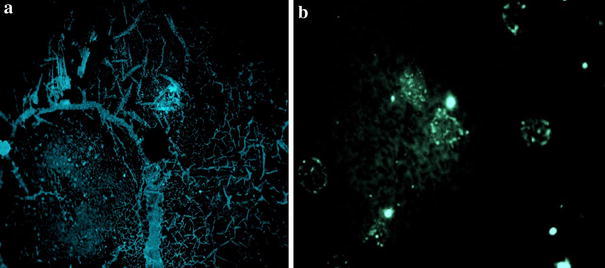
Fluorescent images of F-IgG immobilizations (after successive grafting of PrA and S-IgG) on **a** the polythiophene surface, **b** the gold modified surface

Specific interaction between S-IgG and F-IgG was studied under fluorescence microscopy in order to control the anchoring of S-IgG on the PT modified surface. Figure 9 shows the image of the PT and the gold modified surface.

The images showed fluorescent areas on both surface attesting a specific interaction between the F-IgG and the S-IgG. A non specific adsorption of the F-IgG on the surface would have led to fluorescence on the whole surface. All the recorded images showed a higher fluorescence on the PT surface (Fig. 9a) compared to the gold one (Fig. 9b). This result can not be explained by the light fluorescence of the PT surface (background fluorescence) but indicate a higher coverage rate of S-Ig G on PT compared to gold surface.

The F-IgG deposition was also monitored by QCM and led to mass again of 1,270 ng on polythiophene and 293 ng on gold surface which fully supports the fluorescence conclusions. The corresponding F-IgG/S-IgG ratios are given in Table 1 and indicate that, at the end, the binding capacity of the F-IgG is much higher (close to 2) on the polythiophene surface showing a good compromise between a high number of receptors and a decrease of their accessibility [17].

### *Salmonella* Detection

**Fig. 10:**
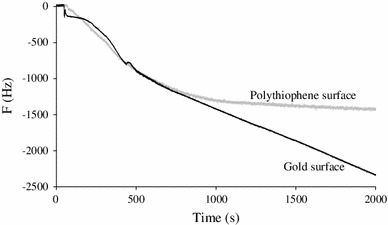
Quartz crystal microbalance responses to *Salmonella* immobilization on both polythiophene and modified gold surfaces (after PrA and S-IgG successive immobilizations)

The QCM measurements during grafting of bacteria on both surfaces are displayed on Fig. 10. On the gold surface, the QCM immobilization curves of the bacteria revealed two stages. The first stage (0–500 s) could be assigned to specific interaction between S-IgG and *Salmonella*, corresponding to antigen recognition. At the end of this stage, the fragments antigen binding (Fab) of S-IgG were almost all occupied by antigens. Then, due to the very high concentration of bacteria in solution, it is probable that the remained bacteria formed clusters and led to non-specific interactions which were observed on the curve after 500 s. On the polythiophene surface, the observed curve exhibited only one stage frequency variation was 1,400 Hz attesting the presence of *Salmonella* recognized by S-IgG.

**Fig. 11:**
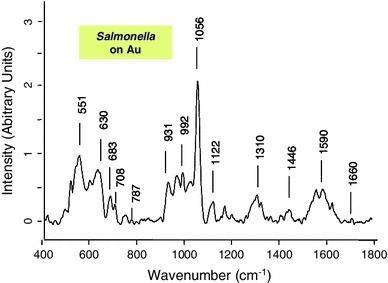
Raman spectra (785 nm, 10 mW) of *Salmonella* immobilized on functionalized gold surface (after successive grafting of PrA and S-IgG)

The bacteria immobilization on the polythiophene did not led to a significantly different raman spectrum compared to those obtained after PrA and S-IgG successive grafting. On gold surface, the spectrum (displayed on Fig. 11) allowed a clear identification of *Salmonella*. As for PrA, the band at 1,446 cm^−1^ could be assigned to the deformation C–H band. The large band at 1,300 cm^−1^ could be attributed to Amid III bond and the band at 1,056 cm^−1^ was attributed to phenylalanine, while tyrosine was detected at 630 cm^−1^ [14, 15]. DNA and RNA are observed at 683 cm^−1^ assigned to guanine, 728 cm^−1^ corresponding to adenine and 787 cm^−1^ corresponding to cytosine and uracil. The last two bands also contain contributions of primary metabolites acetyl-coA and citric acid respectively [29].

## Conclusions

In this article, the ability of two different functionalized surfaces to specifically anchor biomolecules was investigated and an example of *Salmonella* detection was presented. A fast and simple electrochemical procedure was used to synthesize a new polythiophene and the results were compared to those obtained on a similarly modified gold substrate. With this procedure, a chemical linker (*para*-benzene-sulfonyl chloride) successfully covered both substrates and the fast reactivity of this linker with Protein A was also demonstrated leading to high numbers of grafted receptors on the surfaces. The F-IgG/S-IgG ratio was particularly high compared to literature (with SAM) for the polythiophene surface due to a large available surface displayed after polymerization, nevertheless, on this polymer, raman detection of the immobilized biomolecules was made difficult because of signal overlapping. The modified gold surfaces displayed more modest results in term of biomolecule immobilizations (while still in the range of the performances obtained with SAM) but the grafted quantities were far sufficient to allow a fast and easy detection of the bacteria by conventional Raman spectroscopy. This work is an important step for the design of new and cheap biosensors for bacteria detection in all kinds of environments.
